# Supplementary material for: End Sequence Analysis Toolkit (ESAT) expands the extractable information from single-cell RNA-seq data
Source: Genome Res. 2016 Oct;26(10):1397–410. doi: 10.1101/gr.207902.116 (PMC5052061; doi:10.1101/gr.207902.116)
Supplement: Supplemental Material [file supp_gr.207902.116_Supp_Materials.tar.gz › ESAT-esat_v0.1/doc/html/classbroad_1_1core_1_1util_1_1_g_m_t_parser.html]

ESAT: broad.core.util.GMTParser Class Reference


ESAT


- Main Page
- Related Pages
- Packages
- Classes
- Files

- Class List
- Class Index
- Class Hierarchy
- Class Members

All Classes Namespaces Files Functions Variables Enumerator Pages

- broad
- core
- util
- GMTParser

Static Public Member Functions |
List of all members

broad.core.util.GMTParser Class Reference

|  |  |
| --- | --- |
| Static Public Member Functions | |
| static Map< String, Collection  < String > > | ParseGMTFile (File gmtFile, int minNum) throws IOException |
|  | |
| static Map< String, Collection  < String > > | parseGMXFile (File gmxFile, int minNum) throws IOException |
|  | |
| static Map< String, String > | parseCHIPFile (File file) throws IOException |
|  | |
| static void | writeGMT (String save, Map< String, Collection< String >> geneSets) throws IOException |
|  | |
| static Map< String, Collection  < String > > | ParseGeneSetFile (File gmtFile, int minNum) throws IOException |
|  | |
| static Map< String, Collection  < String > > | parseCHIPFileByName (File chipFile) throws IOException |
|  | |

## Member Function Documentation

|  |  |  |  |  |  |
| --- | --- | --- | --- | --- | --- |
| static Map<String, String> broad.core.util.GMTParser.parseCHIPFile | ( | File | *file* | ) | throws IOException |

static

|  |  |  |  |  |  |
| --- | --- | --- | --- | --- | --- |
| static Map<String, Collection<String> > broad.core.util.GMTParser.parseCHIPFileByName | ( | File | *chipFile* | ) | throws IOException |

static

|  |  |  |  |
| --- | --- | --- | --- |
| static Map<String, Collection<String> > broad.core.util.GMTParser.ParseGeneSetFile | ( | File | *gmtFile*, |
|  |  | int | *minNum* |
|  | ) |  | throws IOException |

static

Here is the call graph for this function:

|  |  |  |  |
| --- | --- | --- | --- |
| static Map<String, Collection<String> > broad.core.util.GMTParser.ParseGMTFile | ( | File | *gmtFile*, |
|  |  | int | *minNum* |
|  | ) |  | throws IOException |

static

Here is the caller graph for this function:

|  |  |  |  |
| --- | --- | --- | --- |
| static Map<String, Collection<String> > broad.core.util.GMTParser.parseGMXFile | ( | File | *gmxFile*, |
|  |  | int | *minNum* |
|  | ) |  | throws IOException |

static

Here is the caller graph for this function:

|  |  |  |  |
| --- | --- | --- | --- |
| static void broad.core.util.GMTParser.writeGMT | ( | String | *save*, |
|  |  | Map< String, Collection< String >> | *geneSets* |
|  | ) |  | throws IOException |

static

---

The documentation for this class was generated from the following file:

- java/broad/core/util/GMTParser.java


---

Generated on Fri Aug 15 2014 15:10:57 for ESAT by  

 1.8.7
